# Association of GDNF and CNTNAP2 gene variants with gambling

**DOI:** 10.1556/2006.8.2019.40

**Published:** 2019-08-26

**Authors:** Arundhuti Das, Luca Pagliaroli, Andrea Vereczkei, Eszter Kotyuk, Banrida Langstieh, Zsolt Demetrovics, Csaba Barta

**Affiliations:** 1 Institute of Medical Chemistry, Molecular Biology and Pathobiochemistry, Semmelweis University, Budapest, Hungary; 2 Department of Anthropology, Utkal University, Bhubaneswar, India; 3 Indian Council of Medical Research, Regional Medical Research Center, Bhubaneswar, India; 4 MTA-ELTE Lendület Adaptation Research Group, Institute of Psychology, ELTE Eötvös Loránd University, Budapest, Hungary; 5 Department of Anthropology, North Eastern Hill University, Shillong, India

**Keywords:** addiction, gambling, genetic, GDNF, CNTNAP2

## Abstract

**Background and aims:**

Some form of gambling can be observed in nearly every society, as the gratification felt upon winning in uncertain conditions is universal. A culturally distinct form of gambling, associated with a traditional sporting event of archery known as “*teer*,” is innate to the province of Meghalaya, India. The objective of this study was to find genetic variants underlying this unique form of behavioral addiction. To better understand game-based gambling, we studied genetic variants related to dopaminergic pathways and other genes previously linked to various psychological disorders.

**Methods:**

This study was carried out on a sample of 196 Indo-Aryan adults from Shillong, Meghalaya. Genotyping of glial cell line-derived neurotrophic factor (GDNF) polymorphisms was carried out using real-time PCR. We further investigated 32 single nucleotide polymorphisms located in the 3′ UTR of additional genes of interest using an OpenArray^®^ real-time PCR platform.

**Results:**

Case–control analysis revealed a significant association between GDNF variant rs2973033 (*p* = .00864, χ^2^ = 13.132, *df* = 2) and contactin-associated protein-like 2 (CNTNAP2) variant rs2530311 (*p* = .0448, χ^2^ = 13.132, *df* = 2) with gambling.

**Discussion and conclusions:**

Association of the GDNF gene with gambling could be attributed to its involvement in the development and survival of dopaminergic neurons. Our result is in good agreement with previous data indicating the role of GDNF in certain substance addictions. Several rare variants in the CNTNAP2 gene were also implicated in alcohol addiction in a previous study. This pilot study provides further support for the role of GDNF and CNTNAP2 in addiction behaviors.

## Introduction

Substance-related and addictive disorders are conditions characterized using different classes of drugs that produce reward when used in excess, as defined by the fifth edition of *Diagnostic and Statistical Manual of Mental Disorders* (DSM-5; American Psychiatric Association, 2013). Game-based gambling is considered a prototypical example of behavioral (non-substance) addiction (Robbins & Clark, 2015) in the DSM-5. It is known that about 70%–90% of people gamble at some time in their lives (Shaffer, Hall, & Vander Bilt, 1999; Welte, Barnes, Tidwell, Hoffman, & Wieczorek, 2015); however, only a small portion (1%–2%) will develop a gambling disorder (Xuan et al., 2017).

Gambling involves wagering money or other valuable objects on an event where the outcome is not certain. Thus, gambling implies the willingness to risk something valuable with the hope of a reward. Excessive gambling, however, can lead to negative consequences in subjective well-being by affecting several aspects of daily life, such as mental state, behavior, economy, and relationships. Symptoms are very similar to those observed in substance use dependence (Grant, Potenza, Weinstein, & Gorelick, 2010).

This study was conducted on participants practicing a distinct form of gambling related to the game of archery. In the region of Meghalaya, India, archery (called “*teer*”) is a traditional game among the Khasi population. This sport has been legalized by the local government of Meghalaya and takes place twice a day in the capital city of Shillong. Within a designated arena, a group of professional archers aim and shoot hundreds of arrows at a target. Spectators bid on the last two digits (between 00 and 99) of the total number of arrows that will hit the target. Government-registered counters are spread across the city, and it is here that gamblers buy their tickets with the preferred two-digit number. Following the tournament, individuals who had bid the right guess may collect their prize money from the counters. Despite being a traditional Khasi sport, “*teer*” has gained so much popularity recently that other communities of Shillong also take part in this particular form of game-based gamble. Therefore, this pilot study was performed on a unique, culturally distinct phenotype, where even collection of the sample was a great challenge and as such it should be considered as an exploratory analysis.

Genetic studies of gambling behavior are sparse in literature (Gyollai et al., 2014). In this study conducted on the Indo-Aryan population from Shillong, we mainly focused on glial cell line-derived neurotrophic factor (GDNF), a gene involved in dopaminergic neuronal development, which has previously been found to be associated with methamphetamine (Yoshimura et al., 2011) and tobacco addiction (Kotyuk et al., 2016). In order to investigate the possible role of epigenetic regulation of gene expression in gambling, different single nucleotide polymorphisms (SNPs) located in 3′ untranslated regions (3′ UTRs) of additional candidate genes associated with gambling, such as catechol-O-methyltransferase (Grant, Potenza, Weinstein, & Gorelick, 2015; Guillot, Fanning, Liang, & Berman, 2015; Verdejo-Garcia et al., 2013), DRD2 (Fagundo et al., 2014; Hillemacher et al., 2016; Joutsa et al., 2014), GDNF (Kotyuk et al., 2016; Ron & Janak, 2005), monoamine oxidase A (Ibanez et al., 2000), and solute carrier family 6 member 3 (Fagundo et al., 2014; Kordi-Tamandani, Tajoddini, & Salimi, 2015; Stolf et al., 2014). Some other genes earlier shown to be associated with either addiction, such as HTR2A (Cao et al., 2014; Perez-Rubio et al., 2017), CNR1 (Clarke et al., 2013; Icick et al., 2015), contactin-associated protein-like 2 (CNTNAP2; Song & Zhang, 2014) and TNF (Heberlein et al., 2014), or with other psychiatric conditions, such as AADAC (Bertelsen et al., 2016), ACP1 (Willour et al., 2012), IL1RN (Kapelski et al., 2016), IMMP2L (Petek et al., 2001), LHX6 (Paschou et al., 2012), MEIS1 (Hammerschlag et al., 2017), MIR302A (Beveridge, Gardiner, Carroll, Tooney, & Cairns, 2010), NTN4 (Paschou et al., 2014), and SLITRK1 (Abelson et al., 2005; Speed et al., 2008) were also included. It has been shown that microRNAs (miRNAs) regulate multiple signaling cascades implicated in several types of addictive disorders (Bali & Kenny, 2013; Sim et al., 2017; Smith & Kenny, 2017). Since the 3′ UTR of a gene serves for binding of various miRNAs, a polymorphism in these sequences might alter the binding affinity of regulatory miRNAs to their target messenger RNAs, therefore influencing the expression of the given gene.

We hypothesized that variants potentially affecting miRNA-directed regulation of gene expression may serve as risk factors for behavioral addictions, such as gambling.

## Materials and Methods

### Participants

Samples were collected from individuals residing in Shillong, Meghalaya, located in the North Eastern region of India. The study includes only those individuals whose pedigree can be traced back for three or more consecutive generations. Thus, 196 unrelated adults (130 males, 66 females, mean age: 27.56 ± 9.139 years) from the Indo-Aryan linguistic group were included in the study. Out of the total sample of 196 individuals, 96 were gamblers (49%) and 100 were non-gamblers (51%) and hence were considered as negative control.

### Phenotype measures

The gambling phenotype was determined using the Problem Gambling Severity Index (PGSI). The PGSI classifies the gamblers in four categories: non-problem gambler, low-risk gambler, moderate-risk gambler, and problem gambler. The PGSI was submitted to the participants of the study; the answers were recorded and the gamblers were grouped according to the PGSI parameters. In this study, 5.4% were non-problem gamblers, 12% were low-risk gamblers, 39.1% were moderate-risk gamblers, and 43.5% were problem gamblers. The following parameters were assessed: frequency of gambling, amount of money gambled (from 25 to 15,000 Indian Rupees a day), attitude of the participants, etc. For the statistical analysis in this study, participants were grouped into two categories: gamblers and non-gamblers. The non-gamblers, representing the negative control group, reported no lifetime gambling history.

### The SNP selection criteria

SNPs with a minor allele frequency (MAF) greater than 0.05 were selected from the Single Nucleotide Polymorphism Database (http://www.ncbi.nlm.nih.gov/SNP) of the National Centre for Biotechnology Information. The pairwise tagging method using the linkage disequilibrium r2 threshold of 0.8 by the HaploView Program (Barrett, Fry, Maller, & Daly, 2005) was used to determine tagging SNPs based on HapMap data to obtain a proper coverage of the GDNF gene. In case of a number of options for tagging SNPs, a reference from previous association studies concerning neuropsychiatric disorders was preferred. We selected the following GDNF SNPs: rs3812047, rs11111, rs3096140, rs1549250, and rs2973033.

Table 1 presents the studied SNPs from the 3′ UTR region of additional candidate genes implicated in addiction and other psychiatric disorders. SNPs selection was preceded by *in silico* searches using several miRNA databases to identify SNPs predicted to alter miRNA binding within the 3′ UTR, therefore, more likely to be a risk factor for the phenotype.

**Table 1.** SNPs investigated with the OpenArray genotyping system

Gene	Gene name	SNP
AADAC	Arylacetamide deacetylase	rs1042201
ACP1	Acid phosphatase 1, soluble	rs6855
CNR1	Cannabinoid receptor 1	rs4707436
		rs806368
CNTNAP2	Contactin-associated protein-like 2	rs1062072
		rs2530310
		rs2530311
		rs987456
COMT	Catechol-O-methyltransferase	rs165599
		rs165728
DRD2	Dopamine receptor D2	rs6276
		rs6278
GDNF	Glial cell-derived neurotrophic factor	rs2973051
		rs3749692
		rs62360370
HTR2A	5-Hydroxytryptamine receptor 2A	rs3125
IL1RN	Interleukin 1 receptor antagonist	rs4252041
IMMP2L	Inner mitochondrial membrane peptidase subunit 2	rs1044729
		rs17158195
		rs7795011
LHX6	LIM homeobox 6	rs3750486
		rs74370188
MAOA	monoamine oxidase A	rs3027407
MEIS1	Meis homeobox 1	rs72824830
MIR302A		rs114318553
NTN4	Netrin 4	rs1052651
		rs8699
SLC6A3	Solute carrier family 6 member 3	rs11564774
		rs7732456
SLITRK1	SLIT- and NTRK-like family member 1	rs3737193
		rs41557622
TNF	Tumor necrosis factor	rs3093665

*Note.* SNP: single nucleotide polymorphism.

### Sample preparation and SNP genotyping

Collection of samples and isolation of genomic DNA from buccal swabs were carried out as described previously (Boor et al., 2002). Concentration of the extracted DNA was measured using NanoDrop™ 1000 (Thermo Fisher Scientific, Waltham, MA, USA).

### Genotyping procedure

#### Genotyping of the GDNF gene (real-time PCR)

Genotyping was carried out on the real-time cycler ABI Prism 7300 Real-Time PCR System (Thermo Fisher Scientific). Five SNPs of the GDNF gene located in chromosome position 5p12–p13.1 were genotyped using pre-designed TaqMan^®^ kits with FAM and VIC fluorescent probes: rs3812047 (C_27492935_10), rs11111 (C_8813050_1_), rs1549250 (C_11553504_10), rs3096140 (C_1395038_20), and rs2973033 (C_15958567_10).

#### OpenArray SNPs genotyping

Genotyping of SNPs mentioned in Table 1 was performed on a TaqMan^®^ OpenArray^®^ Genotyping System (Thermo Fisher Scientific). The processing of raw data was performed using TaqMan^®^ Genotyping Software (Thermo Fisher Scientific). We regenotyped 10% of samples as a quality control step to avoid any batch effects. Out of the 32 SNPs considered for assessment, 27 SNPs were successfully genotyped. Out of the 5 SNPs not included in the statistical analysis, three were non-successfully genotyped (rs41557622 of SLITRK1; rs1062072 and rs2530310 of CNTNAP2) and two were found to be non-polymorphic (rs72824830 of MEIS1 and rs4252041 of IL1R).

### Statistical analyses

Standard case–control association analysis was performed using SPSS 20.0 (SPSS Inc., Chicago, IL, USA). The *p* values <.05 were considered nominally significant. Correction for multiple testing was made according to the Bonferroni method: the threshold significance was adjusted to *p* < .0015625 where .0015625 = .05/32 (since 5 + 27 = 32 SNPs, genotyped by the two different methods, were used for the final statistical analysis). We applied the most stringent Bonferroni correction for multiple testing to rule out type I errors in null hypothesis testing. In order to estimate the statistical power of our pilot sample, we performed a post-hoc power calculation using the G*Power software (Faul, Erdfelder, Lang, & Buchner, 2007) to determine the necessary sample size to find significant genetic effects. With a 0.1 effect size (partial eta square value by two groups), by a power set to be at least 0.80, it was determined that 964 participants would be needed in the total sample to detect such an association.

### Ethics

The study protocol was approved by the Departmental Research Committee (DRC) of the School of Human Sciences, North Eastern Hill University. Written informed consent was obtained from all participating individuals.

## Results

### Real-time PCR genotyping results

MAFs of the GDNF SNPs in this study were compared to other Asian population from the HapMap Project (http://www.ncbi.nlm.nih.gov). No significant deviation was observed from the MAFs of Asian population.

A case–control study was conducted to explore possible association of various GDNF gene SNPs with gambling (Table 2). Table 3 shows the results of χ^2^ tests of genotype distribution differences between cases and controls, numbers, and percentages of individuals in each genotype group for (a) non-gamblers (control individuals with no history of gambling) and (b) gamblers. We observed nominally significant differences in case of rs2973033 (*p* = .00027, χ^2^ = 16.391, *df* = 2), rs3812047 (*p* = .00314, χ^2^ = 8.719, *df* = 1) and rs1549250 (*p* = .045, χ^2^ = 6.191, *df* = 2). After correcting for multiple testing, only rs2973033 remained significant (corrected *p* = .00864).

**Table 2.** Location of the real-time PCR-analyzed genetic variants in the vicinity of the GDNF gene

SNP	Location within the gene
rs2973033	5′ UTR
rs3096140	Intron
rs3812047	Intron
rs1549250	Intron
rs11111	3′ UTR

*Note.* SNP: single nucleotide polymorphism; PCR: polymerase chain reaction; GDNF: glial cell line-derived neurotrophic factor; UTR: untranslated region.

**Table 3.** The case–control analysis of any form of gambling and GDNF SNPs

		Non-gamblers		Gamblers				
GDNF SNP	Genotype	*N*	%	*N*	%	*p*	Corrected *p* value	χ^2^
rs2973033	CC	40	40	13	14	.00027**	.00864	16.391
	TT	16	16	22	23.7			
	CT	44	44	58	62.4			
	Total	100		93				
rs3096140	AA	52	60.5	51	56.7	.444	1	1.623
	GG	9	10.5	6	6.7			
	AG	25	29.1	33	36.7			
	Total	86		90				
rs3812047	CC	94	94	75	79.8	.00314*	.10048	8.719
	TT	–	–	–	–			
	CT	6	6	19	20.2			
	Total	100		94				
rs1549250	AA	18	18	13	13.7	.045*	1	6.191
	CC	51	51	36	37.9			
	AC	31	31	46	48.4			
	Total	100		95				
rs11111	CC	8	8.1	4	4.2	.286	1	2.504
	TT	66	66.7	59	62.1			
	CT	25	25.3	32	33.7			
	Total	99		95				

*Note.* χ^2^: chi-square value; *p*: *p* value with .05 significance level; SNP: single nucleotide polymorphism; GDNF: glial cell line-derived neurotrophic factor.

*Nominally significant (*p* < .05). **Significant after multiple correction (*p* < .001).

In case of GDNF SNP rs1549250, the AA and CC genotypes showed higher frequency in case of the non-gamblers (18% and 51%, respectively) compared to those who gambled (13.7% and 37.9%, respectively). In contrast, the frequency of heterozygote AC was higher in individuals who gambled (48.4%) compared to those who never gambled (31%). In case of GDNF SNP rs3812047, the frequency of the CT genotype was higher in gamblers (20.2%) than non-gamblers (6%), suggesting a protective role for the more common CC genotype (the TT homozygous genotype was absent from both groups in our sample). Nevertheless, the above results did not survive Bonferroni correction for multiple testing.

When looking at rs2973033, we observed that the frequency of CT and TT genotypes is higher in case of gamblers (62.4% and 23.7%, respectively) compared to non-gamblers (44% and 16%, respectively). The result suggests that the CT and TT genotypes might serve as risk factors, whereas the CC genotype could be protective against gambling (*p* = .00027, χ^2^ = 16.391, *df* = 2). The association of rs2979033 with gambling remained statistically significant after Bonferroni correction for multiple testing (corrected *p* = .00864).

### OpenArray genotyping results

Table 4 shows the top hits (*p* < .05) of the case–control analysis involving the 3′ UTR SNPs genotyped on the OpenArray platform; detailed results of the successfully genotyped SNPs are available in Supplementary Table S1. SNP rs74370188 in the LHX6 gene showed a nominally significant (*p* = .01571, χ^2^ = 5.834, *df* = 1) association in the χ^2^ test. The frequency of the most prevalent GG genotype is higher in non-gamblers (78.0%) than in gamblers (61.7%). However, rs74370188 does not survive the Bonferroni correction for multiple testing.

**Table 4.** The top two SNPs (*p* < .05) of the case–control analysis of any form of gambling and SNPs in 3′ UTR: OpenArray results

		Non-gamblers		Gamblers					
	Genotype	*N*	%	*N*	%	*p*	Corrected *p* value	χ^2^	OR
*CNTNAP2*									
rs2530311	AA	21	23.9	41	47.7	.00140**	.0448	13.132	NA
	GG	19	21.6	7	8.1				
	AG	48	54.5	38	44.2				
	Total	88		86					
*LHX6*									
rs74370188	AA	–	–	–	–	.01571*	.5072	5.834	0.454
	GG	71	78	58	61.7				
	AG	20	22	36	38.3				
	Total	91		94					

*Note.* χ^2^: Chi-square value; OR: odds ratio; UTR: untranslated region; SNP: single nucleotide polymorphism; NA: not applicable; *p*: *p* value with .05 significance level.

*Nominally significant (*p* < .05). **Significant after multiple correction (*p* < .001).

In the case of rs2530311 located in CNTNAP2 gene, we observed a significant association (*p* = .00140, χ^2^ = 13.132, *df* = 2) in the χ^2^ test, which withstands correction for multiple testing (corrected *p* = .0448). The frequency of the AA genotype of rs2530311 is higher in case of gamblers (47.7%) compared to non-gamblers (23.9%). Thus, the AA genotype seems to be the risk factor for gambling.

## Discussion

This study was conducted on a population of gamblers involved in a local archery game in Meghalaya, India. The traditional sport, commonly known as “teer,” has gained widespread popularity among locals of Shillong in part due to the high odds offered by the organizers, allowing winners to take home 80 times the sum that was gambled.

Our case–control genetic analysis identified two significant associations between gambling and the GDNF gene SNP rs2973033 (*p* = .00864, χ^2^ = 16.391, *df* = 2) and the CNTNAP2 gene SNP rs2530311 (*p* = .0448, χ^2^ = 13.132, *df* = 2) after correction for multiple testing.

GDNF is a well-studied gene associated with addiction and plays a role in several processes including drug reward, drug self-administration, relapse, and abstinence (Ghitza et al., 2010; Wise, 2004). The gene encodes a secreted ligand of the transforming growth factor-beta (TGF-β) receptor superfamily of proteins. Upon ligand binding to various TGF-β receptors, transcription factors of the SMAD family are recruited and activated, resulting in gene expression changes. GDNF is involved in the growth, survival, and morphological differentiation of dopaminergic neurons during development as well as enhanced high-affinity uptake of dopamine in rat embryonic midbrain cells (Lin, Doherty, Lile, Bektesh, & Collins, 1993). The role of GDNF in reward mechanisms has been shown in animal model studies with drugs like cocaine, methamphetamine, and alcohol (Ghitza et al., 2010). Moreover, external injection of GDNF in rats was shown to reduce the impact of dopamine depletion observed during drug abstinence (Barak, Carnicella, Yowell, & Ron, 2011). A GDNF knockout study in mice has also shown increased sensitivity for alcohol in case of heterozygotes compared to wild-type homozygotes, thus confirming the role of endogenous GDNF in reducing the rewarding effects of ethanol (Carnicella, Ahmadiantehrani, Janak, & Ron, 2009). In a previous study conducted by our group, strong association was observed between GDNF SNPs rs3812047 and rs11111 with nicotine and betel quid use (unpublished data).

In this study, we report a significant association of rs2973033 in GDNF with game-based gambling. Our results confirm the role of GDNF in addiction, and to our knowledge, this is the first study to show association of GDNF gene polymorphisms with gambling as a type of behavioral addiction.

The CNTNAP2 gene codes for a neuronal transmembrane protein belonging to the neurexin superfamily involved in communication between neuronal cells (Condro & White, 2014). This protein is also involved in localization of potassium channels within differentiating axons (Poliak et al., 1999). The gene encompasses almost 1.5% of chromosome 7 and is one of the largest genes in the human genome. Due to its large extent, it is likely a target for structural rearrangements, including copy number variations, translocations, altered transcription factor binding, and also epigenetic modifications, including miRNA regulation. The CNTNAP2 gene has been implicated in several neurodevelopmental disorders. Due to a translocation affecting the coding region of the gene, it was found to be associated with Gilles de la Tourette syndrome, intellectual disability, and obsessive–compulsive disorder (Verkerk et al., 2003). Other cognitive disorders, such as schizophrenia, epilepsy, autism, and attention-deficit hyperactivity disorder, were also linked to disruptions in CNTNAP2 gene (Rodenas-Cuadrado, Ho, & Vernes, 2014). SNP variants of this gene were also linked to various complex neurological disorders including autism (Alarcon et al., 2008; Arking et al., 2008; Strauss et al., 2006), schizophrenia, and depression (Ji et al., 2013). CNTNAP2 gene was previously identified in alcohol addiction (Song & Zhang, 2014). The study used a new computational method, tree-based analysis of rare variants, to investigate the effect of rare variants in complex disorders. The authors suggested that 97 rare variants of the CNTNAP2 gene could be protective against alcohol addiction in women. In this study, we found that the common variant rs2530311 of the CNTNAP2 gene is associated with gambling, thus corroborating the involvement of this gene in the addiction behavior.

The main limitation of the study is the relatively small sample size. However, this is a pilot study performed with a very unique, culturally distinct phenotype and even this collection has proven to be a great challenge. Thus, our final sample consisted of 196 participants, on which the exploratory genetic association analysis was conducted.

In conclusion, to our knowledge, this is the first study reporting a direct link between CNTNAP2 and game-based gambling. This study further confirms the role of GDNF in addiction, while it is the first study to show the association of GDNF polymorphism with gambling as a type of behavioral addiction.
